# Targeting Mitochondrial ROS-Mediated Ferroptosis by Quercetin Alleviates High-Fat Diet-Induced Hepatic Lipotoxicity

**DOI:** 10.3389/fphar.2022.876550

**Published:** 2022-04-12

**Authors:** Jin-Jin Jiang, Guo-Fu Zhang, Jia-Yi Zheng, Ji-Hu Sun, Shi-Bin Ding

**Affiliations:** ^1^ Jiangsu Vocational College of Medicine, Yancheng, China; ^2^ Department of Public Health, Xinxiang Medical University, Xinxiang, China

**Keywords:** quercetin, mitochondrial ROS, ferroptosis, nonalcoholic fatty liver disease, hepatic lipotoxicity

## Abstract

**Background:** The protective effect of quercetin on nonalcoholic fatty liver disease (NAFLD) has been reported, but its mechanism remains poorly understood. Recently, quercetin was reported to be capable of inhibiting ferroptosis, which is a recognized type of regulated cell death. Moreover, hepatic ferroptosis plays an important role in the progression of NAFLD, but experimental evidence is limited. Hence, our study aimed to investigate the effect of quercetin on hepatic ferroptosis in high-fat diet (HFD)-induced NAFLD and further elucidate the underlying molecular mechanism.

**Methods:** C57BL/6J mice were fed either a normal diet (ND), an HFD, or an HFD supplemented with quercetin for 12 weeks. Hepatic lipid peroxidation, steatosis, ferroptosis and iron overload were examined. *In vitro*, steatotic L-02 cells was used to study the potential mechanism.

**Results:** We found that the HFD caused lipid peroxidation, lipid accumulation and ferroptosis in the liver, which were rescued by quercetin supplementation. Consistent with the *in vivo* results, quercetin alleviated lipid droplet accumulation and reduced the levels of lipid reactive oxygen species (ROS) and ferroptosis in steatotic L-02 cells. Using a mitochondrial ROS (MtROS) scavenger (Mito-TEMPO) and ferroptosis specific inhibitor (Fer-1), we found that quercetin remarkably alleviated lipid droplet accumulation and lipid peroxidation by reducing MtROS-mediated ferroptosis in steatotic L-02 cells.

**Conclusion:** Our data showed that HFD consumption induced lipid accumulation and triggered ferroptosis in liver, ultimately leading to hepatic lipotoxicity, which can be alleviated by quercetin. Findings from this study provide new insight into the mechanism by which quercetin can be used for the prevention and treatment of NAFLD.

## Introduction

Nonalcoholic fatty liver disease (NAFLD) is a spectrum of chronic liver diseases that includes simple hepatic steatosis (NAFL), nonalcoholic steatohepatitis (NASH), fibrosis and cirrhosis, and its incidence continues to increase worldwide. It has been indicated that consumption of a high-fat diet (HFD) for extended periods of time leads to NAFLD (Kim et al., 2016). At present, the prevalence of NAFLD worldwide is estimated to be approximately 25%, and NAFLD has become a major public health issue (Younossi et al., 2019). Steatosis is characterized by excess triglyceride accumulation stored as lipid droplets in the cytosol of hepatocytes (Willebrords et al., 2015), which is deemed the first stage of NAFLD. Hepatic steatosis may develop into NASH, fibrosis, cirrhosis and eventually hepatocellular carcinoma without timely interventions (Angulo, 2002).

The “two-hit hypothesis” is a widely accepted mechanism explaining the pathogenesis of NAFLD (Day and James, 1998; Rada et al., 2020). According to the hypothesis, hepatic steatosis (accumulation of excess triglycerides in hepatocytes) serves as the “first hit”, and oxidative stress, lipid peroxidation and inflammation act as the “second hit”. In addition, the “multiple hit” hypothesis considers multiple factors that synergistically cause the development of NAFLD (Tilg and Moschen, 2010), including oxidative stress and lipid peroxidation. Thus, the inhibition of oxidative stress and lipid peroxidation is an effective NAFLD treatment strategy. In addition to decreased energy intake and increased energy expenditure, antioxidant supplementation is one of the most recommended methods for the prevention and amelioration of NAFLD. Quercetin (3,3,4,5,7-pentahydroxyflavone, Que) is a flavonoid that is abundant in vegetables, fruits, herbs and teas and exhibits antioxidant and anti-inflammatory activities (Anand David et al., 2016). Mounting laboratory evidence *in vitro* and *in vivo* suggests that Que has beneficial effects on HFD-induced NAFLD (Ferramosca et al., 2017). The possible mechanisms include the promotion of hepatic very low-density lipoprotein assembly and lipophagy (Zhu et al., 2018); the amelioration of inflammation, oxidative stress and lipid metabolism (Zhu et al., 2018); and the mediation of intestinal microbiota imbalance and activation of its related gut-liver axis (Porras et al., 2017; Yang et al., 2019). The potential mediative effect of Que on ferroptosis to protect against NAFLD has not been studied.

Ferroptosis is a recently recognized nonapoptotic form of regulated cell death that is characterized by iron-dependent lipid peroxidation (Dixon et al., 2012). The regulatory pathways of ferroptosis includes three main biological axes: 1) glutathione/glutathione peroxidase 4 (GSH/GPX4) pathway, 2) lipid metabolism pathways, 3) iron metabolism-regulated ferroptosis pathway (Capelletti et al., 2020; Jiang et al., 2021). Ferroptosis is regulated by integrated oxidation and antioxidant systems in cells (Chen et al., 2020), which play vital roles in the pathological processes of NAFLD (Li X. et al., 2020; Qi et al., 2020). Recently, ferroptosis was confirmed to be the initial cell death process that triggers NASH (Tsurusaki et al., 2019), but whether it is involved in HFD-induced NASH is not certain. Que has been reported to significantly decrease hepatic iron and effectively quench free iron-induced hydroxyl radical production in hepatocytes (Oliva et al., 2011). A previous study found that Que alleviated type 2 diabetes by inhibiting pancreatic iron deposition and pancreatic β cell ferroptosis (Li D. et al., 2020). In an *in vitro* study, Que also had a beneficial effect on acute kidney injury by inhibiting ferroptosis (Wang et al., 2021). However, its inhibitory effect on ferroptosis in NAFLD has not been reported. Therefore, we hypothesized that Que exerts a protective effect on NAFLD by inhibiting hepatic ferroptosis to thereby reduce hepatic lipotoxicity.

In our current study, we confirmed that mitochondrial reactive oxygen species (MtROS) were involved in ferroptosis in NASH. Moreover, we demonstrated that Que reduced lipid peroxidation, fat accumulation, inflammation and iron overload and inhibited ferroptosis in hepatocytes and the livers of HFD-fed mice and that this effect was associated with reduced MtROS in hepatocytes and thus protected against NAFLD. This study provides new insight into the protective effects of Que against NAFLD.

## Materials and Methods

### Reagents and Antibodies

Que (3,3,4,5,7-pentahydroxyflavone, purity ≥95%) was purchased from Solarbio (Beijing, China). Fetal bovine serum (FBS) and DMEM were obtained from Gibco (United States). The primary antibodies, including anti-glutathione peroxidase 4 (GPX4), anti-cyclooxygenases-2 (COX2), anti-ACSL4, anti-GAPDH, and IgG (H + L)-HRP secondary antibodies were purchased from Proteintech Bio, Inc. (Wuhan, China).

### Animal Care and Experimental Design

Six-week-old male C57BL/6J mice (18–22 g) were obtained from Vital River Laboratory Animal Technology Co., Ltd. (Beijing, China). Mice were maintained under specific pathogen-free conditions at 23 ± 2°C and a 12-h light/dark cycles and had free access to food and water. All animal protocols were approved by the Animal Care and Use Committee of the Xinxiang Medical University.

After 1  week of adaptive feeding, all mice were randomly divided into the following four groups (*n* = 10 per group): 1) the normal diet (ND) group, mice fed a ND (18% calories from fat; TROPHIC Animal Feed High-tech Co., Ltd., China); 2) the HFD group, mice fed a HFD (45% calories from fat; TROPHIC Animal Feed High-tech Co., Ltd., China) for 12 weeks when the experiment beginning; 3) the HFD-Que/L group, mice fed a HFD supplemented with 50 mg/kg body weight; and 4) the HFD-Que/H group, mice fed a HFD supplemented with 100 mg/kg body weight. After the experiment beginning, mice in the Que-treated groups were administered Que (50 and 100 mg/kgbw) *via* orally gavage daily for 12 weeks, and mice in the ND and HFD groups were administered deionized water at the same time *via* oral gavage. After treatment, the mice were sacrificed by intravenous pentobarbital injection (20 mg/kg). Blood samples were obtained by cardiac puncture to separate the serum. Liver tissues were quickly removed, weighed and then fixed in 4% formaldehyde solution for histopathologic examination or snap-frozen in liquid nitrogen and stored at −80°C until use.

### Cell Culture and Cell Viability Assay

L-02 cells were incubated at 37°C and 5% CO_2_ in Dulbecco’s modified Eagle’s medium (DMEM) supplemented with 10% fetal bovine serum, 100 U/ml penicillin and 100 μg/ml streptomycin. Cell viability was assessed by the Cell Counting Kit-8 (CCK-8) assay (Beyotime Biotechnology, Shanghai, China). L-02 cells were treated with Que for 24 h, after which CCK-8 reagent was added to the cell wells, and the absorbance was determined at 450 nm.

### Biochemical Measurements

Serum alanine aminotransferase (ALT) and aspartate aminotransferase (AST) levels were determined using commercial enzyme kits (Nanjing Jiancheng Bioengineering, Inc., Nanjing, China). Hepatic total lipids were extracted as previously described (Loison et al., 2002), and the levels of total cholesterol (TC) and total triglycerides (TG) in livers were then measured using commercial assay kits (Sangon Biotech, Inc., Shanghai, China) according to the manufacturer’s protocols.

### Iron Assay

Iron content in liver tissue and cells was determined by a colorimetric assay kit according to the manufacturer’s instructions (MLBIO, Inc., Shanghai, China).

### Histopathologic Examination for Livers

Frozen liver sections from OCT-embedded liver samples were stained with Oil Red O to assess fat accumulation. Liver samples were fixed in 4% paraformaldehyde for 24 h at room temperature and embedded in paraffin, and 5 μm-thick sections were then stained with hematoxylin-eosin (H&E) for histological assessment. A point-counting method was used for calculating hepatic steatosis as previously described (Catta-Preta et al., 2011; Xu et al., 2017).

### Steatotic Hepatocyte Model

To establish the MD, L-02 cells at 80% confluency were incubated with a 0.5 mmol/L free fatty acid (FFA) mixture (Kunchuang Biotechnology, Xi’an, China) for 24 h and then used for further experiments. Palmitic acid (PA) was coupled to palmitate-free bovine serum albumin (BSA) in a ratio of 2 mM PA: 3% BSA in a 40°C water bath to prepare the FFA mixture.

### Quantification of TG in L-02 Cells

To evaluate intracellular TG content, L-02 cells were scraped. Then TG content was determined using a commercial assay kit (Sangon Biotech, Inc., Shanghai, China) according to the manufacturer’s protocols.

### Ferroptosis Inhibitor Treatment

Before treatment with the FFA mixture, L-02 cells were pretreated with the ferroptosis specific inhibitor ferrostatin-1 (Fer-1, 10 μmol/L) (APEx Bio, United States) for 4 h.

### Measurement of Oxidative Stress

The contents of reduced glutathione (GSH)/GSSG (oxidized glutathione) in the liver tissues of mice and cell lysates were measured by commercial assay kits (Nanjing Jiancheng Bioengineering, Inc., Nanjing, China) according to the manufacturers’ instructions. The level of 4-hydroxynonenal (4-HNE) in the liver tissues of mice and cell lysates was determined with a commercial ELISA kit (MLBIO, Inc., Shanghai, China).

### Oil Red O Staining of Hepatocytes

To observe the lipid droplets in hepatocytes, L-02 cells were incubated in six-well plates, treated as described, washed with phosphate-buffered saline three times, fixed with 4% paraformaldehyde for 30 min, and stained with Oil red O for 15 min at room temperature. A microscope connected to a digital camera (Olympus, Tokyo, Japan) was used to observe and capture lipid droplets in L-02 cells.

### Measurement of Mitochondrial Reactive Oxygen Species

The L-02 cells were pretreated with Que (50 μmol/L) or mito-TEMPO (60 μmol/L) (APEx Bio, United States) for 4 h and then incubated with the FFA mixture for 24 h to establish the steatotic hepatocyte model. After the experimental treatment, L-02 cells were incubated in 5 μmol/L MitoSox Red Mitochondrial Superoxide indicator (Thermo, United States) in phosphate buffered saline at 37°C for 30 min in the dark. The cells were washed with warm phosphate-buffered saline three times and then immediately analyzed by flow cytometry.

### Flow Cytometric Analysis of Lipid Reactive Oxygen Species

The L-02 cells were pretreated with Que (25 and 50 μmol/L) for 4 h and then incubated with the FFA mixture for 24 h to establish the steatotic hepatocyte model. Cells were incubated with the Molecular Probes BODIPY 581/591C11 (Invitrogen, United States) working solution (5 μmol/L) at 37°C for 30 min in the dark. The cells were washed with phosphate buffered saline three times and measured using flow cytometric analysis.

### Western Blotting

Total proteins were extracted from liver tissues and L-02 cells using RIPA lysis buffer protein extraction reagent (key GEN, Nanjing, China). The protein concentrations of the samples were measured using a BCA protein assay kit (DINGGUO, Bio Co., Ltd., Beijing, China). Protein samples were electrophoresed on polyacrylamide gels and then transferred onto polyvinylidene difluoride (PVDF) membranes. The membranes were blocked with 5% nonfat milk in PBS and incubated with primary antibodies against GPX4, COX-2, ACSL4, and GAPDH overnight at 4°C, followed by incubation with the corresponding secondary antibody for 1 h. The protein bands were detected using an ECL detection system (Tanon, Shanghai, China). The relative intensities of the target bands were quantified by Image Studio Software (Li-Cor Biosciences) and normalized to the optical density of GAPDH.

### Statistical Analysis

Data are expressed as the mean ± standard deviation (SD). All data were analyzed with SPSS 25.0 (SPSS, Chicago, IL, United States). Comparisons of groups were analyzed using one-way ANOVA followed by post hoc analysis (Bonferroni posttest), and a *p* value less than 0.05 was considered to indicate statistical significance.

## Results

### Effects of Que on Biochemical Parameters and Hepatic Lipid Accumulation in High-Fat Diet-Fed Mice

**FIGURE 1:**
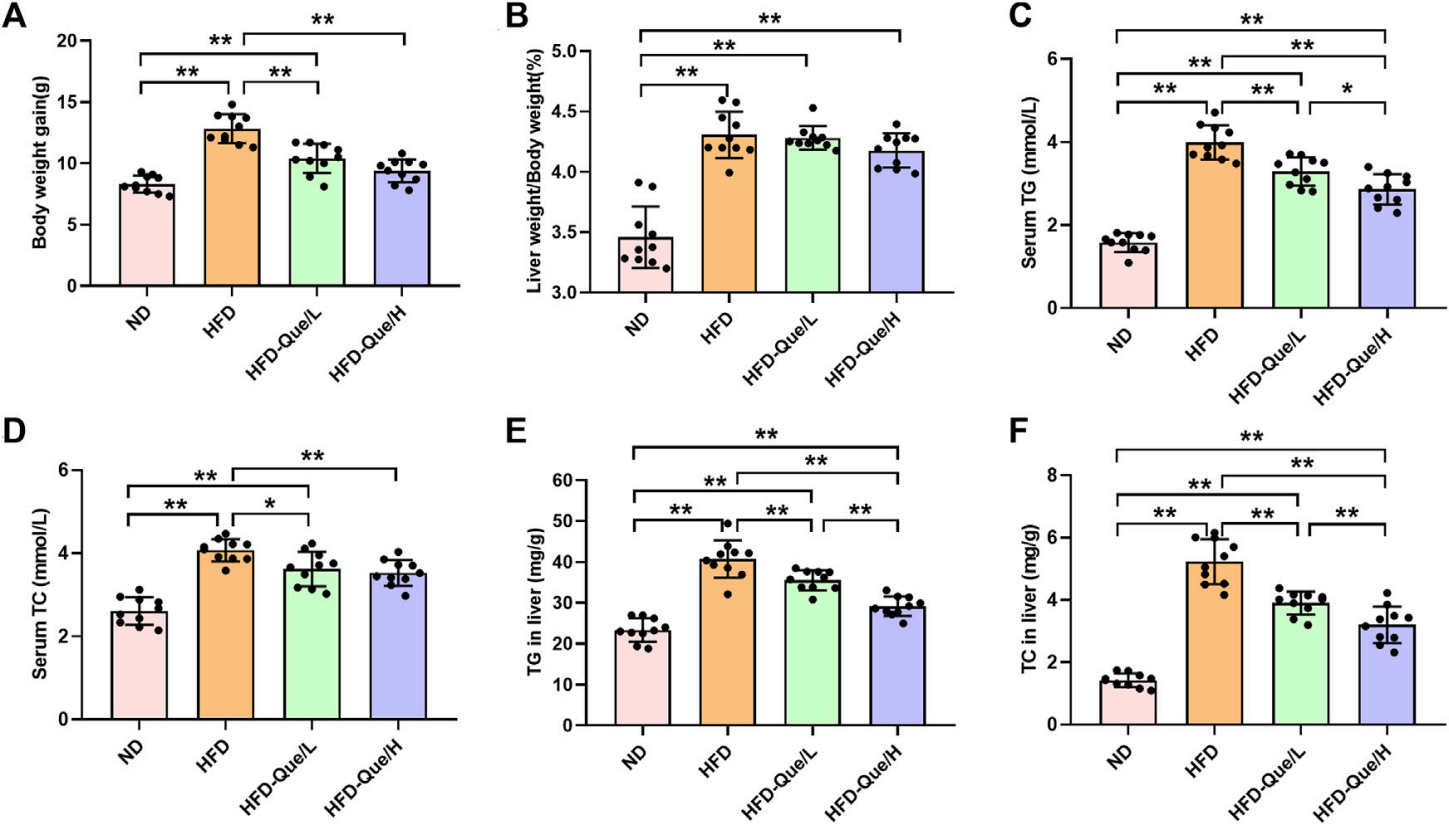
Que alleviated lipid metabolism parameters in HFD-fed mice (*n* = 10). **(A)** Body weight gain. **(B)** Liver weight/Body weight. **(C)** Serum TG. **(D)** Serum TC. **(E)** TG in the liver. **(F)** TC in the liver. The data are expressed as the mean ± SD. **, *p* < 0.01 and *, *p* < 0.05.

**FIGURE 2:**
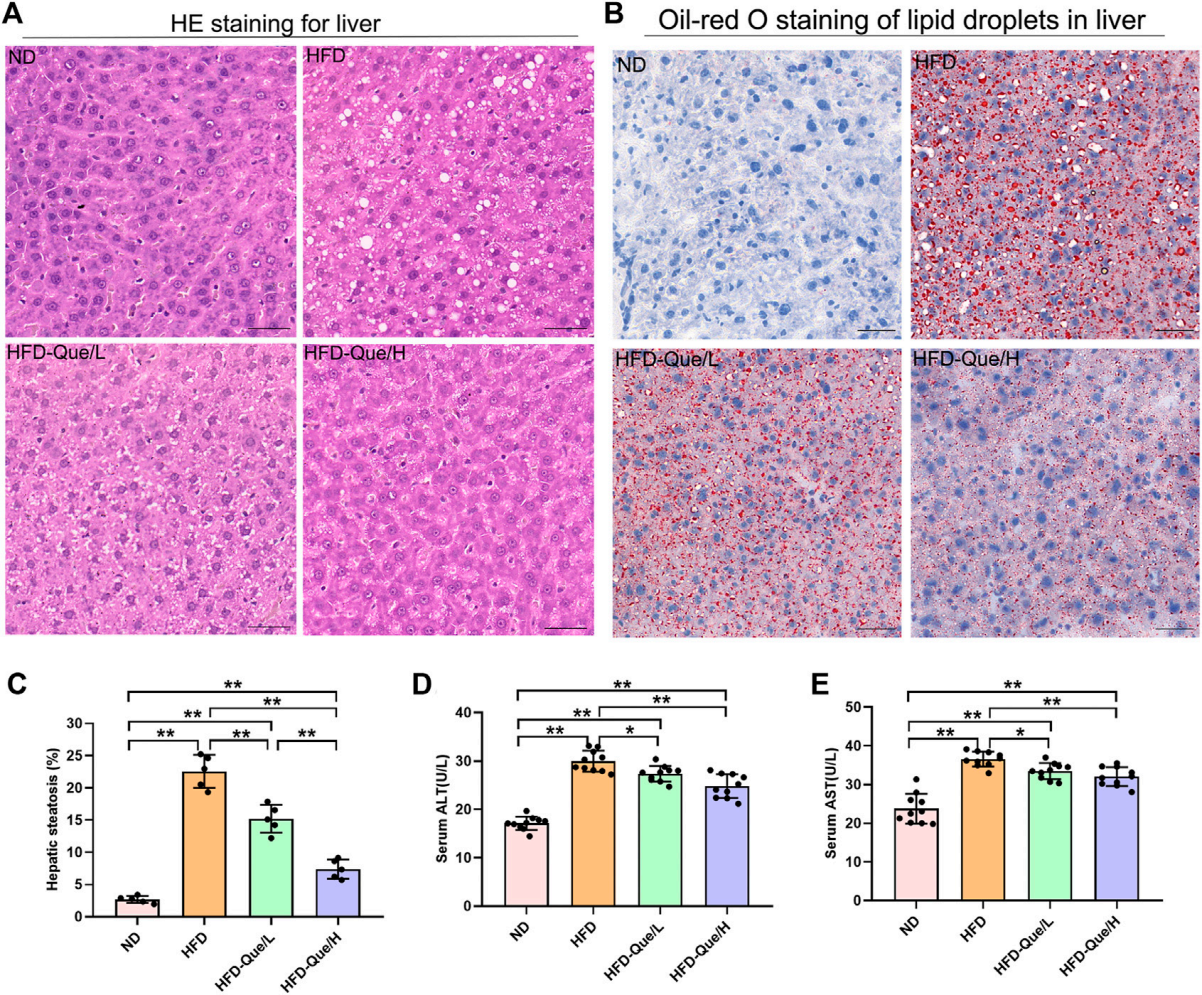
Que alleviated lipid accumulation and hepatic injury in HFD-fed mice. **(A)** Oil Red O staining of the liver (×200, scale bars = 100 μm). **(B)** H&E staining of the liver (×200, scale bars = 50 μm). **(C)** The quantitation of hepatic steatosis (*n* = 5). **(D)** Serum ALT (*n* = 10). **(E)** Serum AST (*n* = 10). The data are expressed as the mean ± SD. **, *p* < 0.01 and *, *p* < 0.05.

As shown in Figures 1A,B, HFD-fed mice showed increased body weight gains and the ratio of liver weight/body weight compared to those of mice in the ND group, and administration of Que significantly alleviated the increases in body weight gain caused by the HFD. To assess the lipid metabolism status after HFD feeding, the levels of TG and TC in the serum and liver were measured (Figures 1C–F). Compared to the ND group, the serum and hepatic levels of TG and TC were markedly elevated in the HFD group. Moreover, Que supplementation attenuated the TG and TC levels in HFD-fed mice in a dose-dependent manner. Liver sections stained with H&E and Oil Red O were used to evaluate the liver histopathological changes in the four groups. As shown by H&E staining, the occurrence of hepatic steatosis was significantly higher in the HFD-fed mice than in the mice of the ND group (Figure 2A). Consistent with the results of hepatic steatosis, mice in the HFD group showed more lipid droplet accumulation than those in the ND group (Figure 2B). Furthermore, the hepatic steatosis percentage and lipid droplets were much lower in the HFD-Que/L and HFD-Que/H group mice than in mice of the HFD group (Figure 2C). In addition, the serum levels of AST and ALT were increased in the mice of the HFD group compared with the ND group, and the elevated serum AST and ALT levels in the HFD-fed mice were alleviated by Que supplementation to varying degrees (Figures 2D,E).

### Que Alleviated Hepatic Lipid Peroxidation in High-Fat Diet-Fed Mice

**FIGURE 3:**
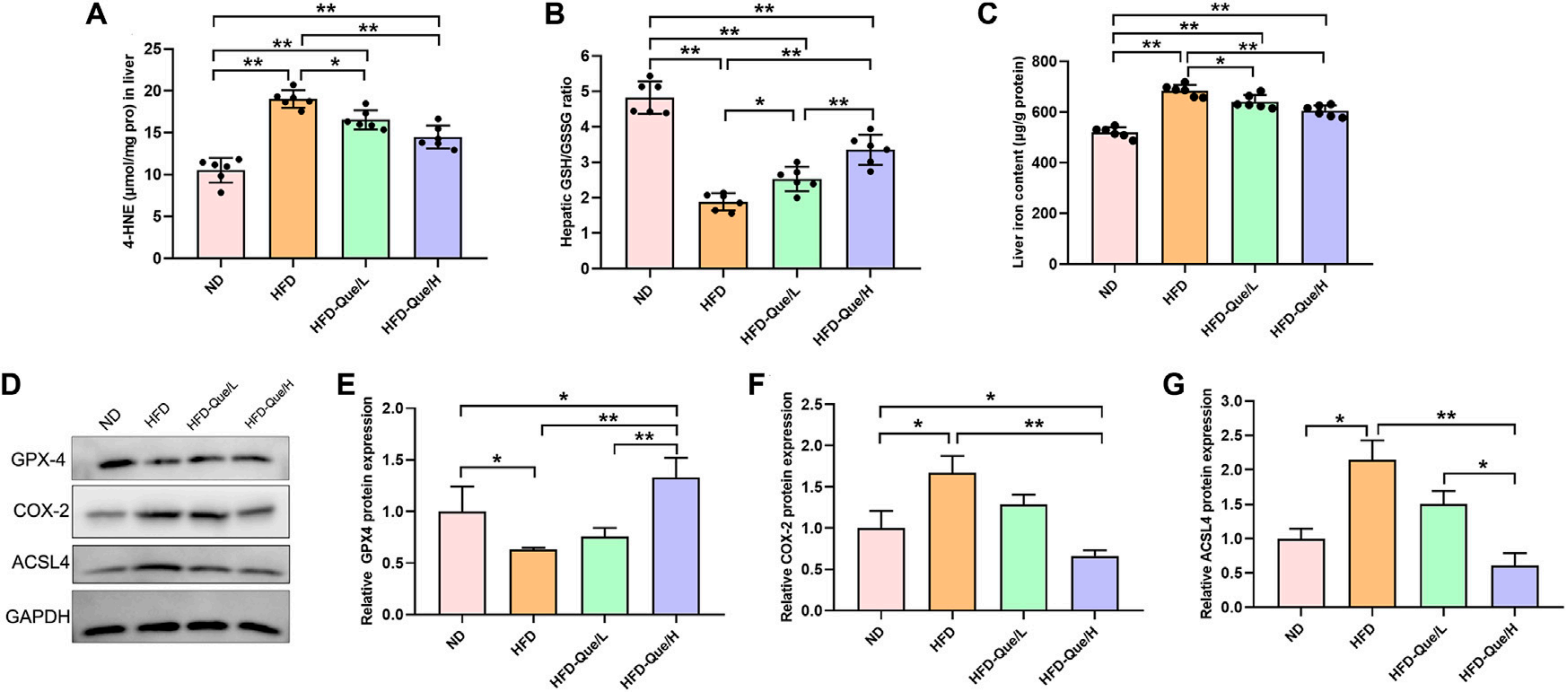
Que reduced lipid peroxidation and ferroptosis in the livers of HFD-fed mice. **(A,B)** The levels of 4-HNE and GSH/GSSG in the liver (*n* = 6). **(C)** Iron content in the liver (*n* = 6). **(D)** Representative Western blot images. **(E–G)** Quantitative analysis of ferroptosis-related proteins (GPX4, COX-2 and ACSL4) in the liver (*n* = 3). The data are expressed as the mean ± SD. **, *p* < 0.01 and *, *p* < 0.05.

To explore whether Que alleviated HFD-induced hepatic lipid peroxidation *in vivo*, we measured the levels of 4-HNE and GSH/GSSG (Figures 3A,B). Compared with those in the ND group, the concentration of 4-HNE was obviously upregulated and the ratio of GSH/GSSG was obviously downregulated in the HFD group. However, the concentrations of 4-HNE in the HFD-Que/L and HFD-Que/H groups were significantly lower than that in the HFD group. Moreover, the ratio of GSH/GSSG in the Que-treated mice was significantly higher than that in the HFD group. These data indicated that Que has a protective effect on HFD-induced hepatic lipid peroxidation.

### Que Decreased Liver Iron Content and Inhibited Hepatic Ferroptosis in High-Fat Diet-Fed Mice

To study whether Que has an inhibitory effect on hepatic ferroptosis *in vivo*, we determined the iron content and the protein expression of GPX4, COX-2, and ACSL4 in liver tissues (Figures 3C–G). Compared to the ND group, the liver iron content was markedly increased in the HFD group, and the elevated liver iron content in the HFD-fed mice were alleviated by Que supplementation (Figure 3C). Compared with that in the ND group, GPX4 expression was significantly downregulated in the HFD group, while COX-2 and ACSL4 expression was significantly higher in the HFD group than in the ND group (Figures 3D–G). Moreover, Que supplementation markedly increased GPX4 protein expression and obviously decreased the COX-2 and ACSL4 protein expression in liver tissues (Figures 3D–G). These data indicated that long-term HFD treatment induced hepatic ferroptosis *in vivo*, which was rescued by Que supplementation.

### Que Suppressed Lipid Droplet Accumulation and Lipid Reactive Oxygen Species in the Steatotic Hepatocyte Model

**FIGURE 4:**
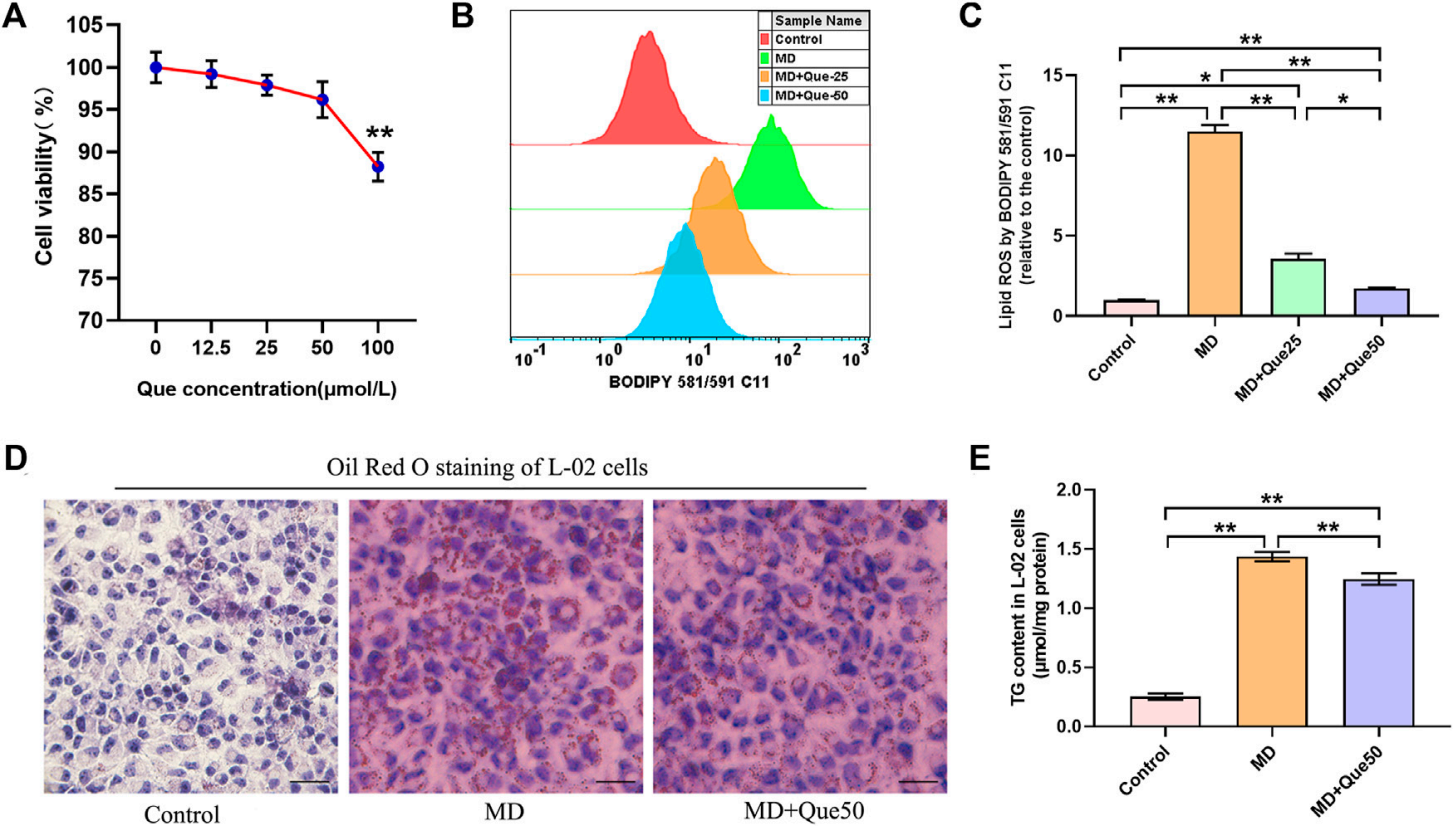
Que reduced lipid ROS and lipid droplet accumulation in steatotic L-02 cells (*n* = 3). **(A)** The effect of Que treatment on L-02 cell viability. **(B)** Representative fluorescence intensity images of cellular lipid ROS by BODIPY 581/591 C11 obtained by flow cytometry. **(C)** Flow cytometric analysis of fluorescence intensity. **(D)** Oil Red O staining of L-02 cells (scale bar = 50 μm). **(E)** TG content in L-02 cells. The data are expressed as the mean ± SD from three independent experiments. **, *p* < 0.01 and *, *p* < 0.05.

The viability of L-02 cells exposed to Que was evaluated by CCK-8 assays. L-02 cells were treated with different doses of Que (0–100 μmol/L) for 24 h. The viability of L-02 cells treated with Que showed a slight decline at the high dose of Que (100 μmol/L) (Figure 4A). The flow cytometry results showed that lipid ROS accumulated in steatotic L-02 cells, and Que treatment reduced the production of lipid ROS in steatotic L-02 cells (Figures 4B,C). As shown in Figures 4D,E, lipid accumulation in the MD group was significantly higher than that in the control group. Compared with the MD group, Que (50 μmol/L) decreased the lipid accumulation in steatotic L-02 cells (Figures 4D,E).

### Que Inhibited Hepatic Lipid Peroxidation and Ferroptosis in Steatotic Hepatocytes

**FIGURE 5:**
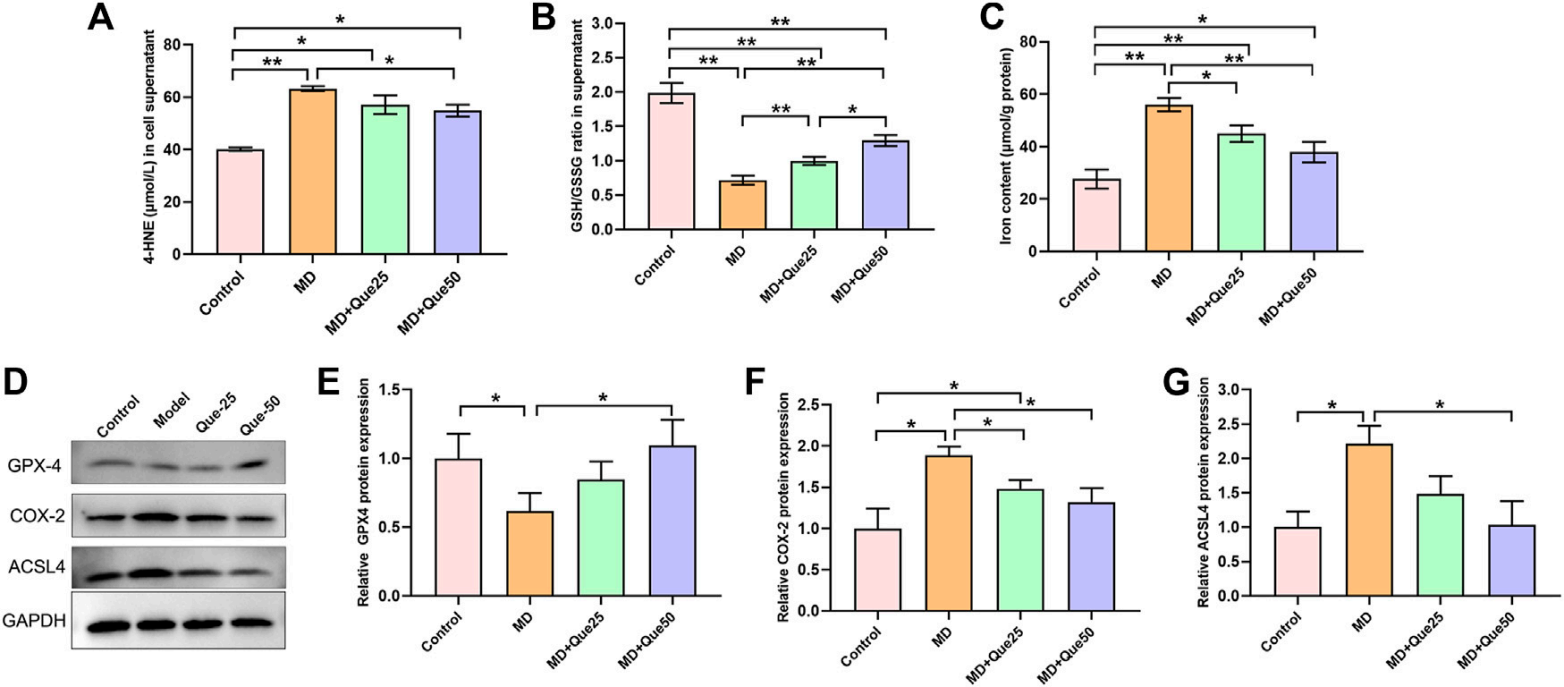
Que suppressed lipid peroxidation and ferroptosis in steatotic L-02 cells. **(A,B)** The relative contents of 4-HNE and GSH/GSSG in L-02 cells (*n* = 3). **(C)** Iron content in cells (*n* = 3). **(D)** RepresentativeWestern blot images. **(E–G)** The expression of ferroptosis-related proteins (GPX4, COX-2 and ACSL4) in L-02 cells (*n* = 3). The data are expressed as the mean ± SD from three independent experiments. ∗∗*p* < 0.01 and ∗*p* < 0.05.

The content of 4-HNE was apparently increased and the ratio of GSH/GSSG was decreased in the MD group compared with the control group (Figures 5A,B). Moreover, the increase in 4-HNE and the decrease in the GSH/GSSG ratio in steatotic hepatocytes were reduced by Que treatment (Figures 5A,B). The cellular iron content in the MD group was higher than the control group, and elevated cellular iron content in steatotic hepatocytes was decreased by Que treatment (Figure 5C). As shown in Figures 5D–G, compared with those in the control group, the protein expression of GPX4 was downregulated and the protein expression of COX-2 and ACSL4 was upregulated in the MD group. Additionally, both the decreased GPX4 expression and the increased COX-2 and ACSL4 expression in the MD group were rescued by Que treatment. Taken together, these data suggest that Que inhibits ferroptosis in steatotic hepatocytes.

### Que Inhibited Ferroptosis by Reducing Mitochondrial Reactive Oxygen Species and Decreased Lipid Deposition in Steatotic Hepatocytes

**FIGURE 6:**
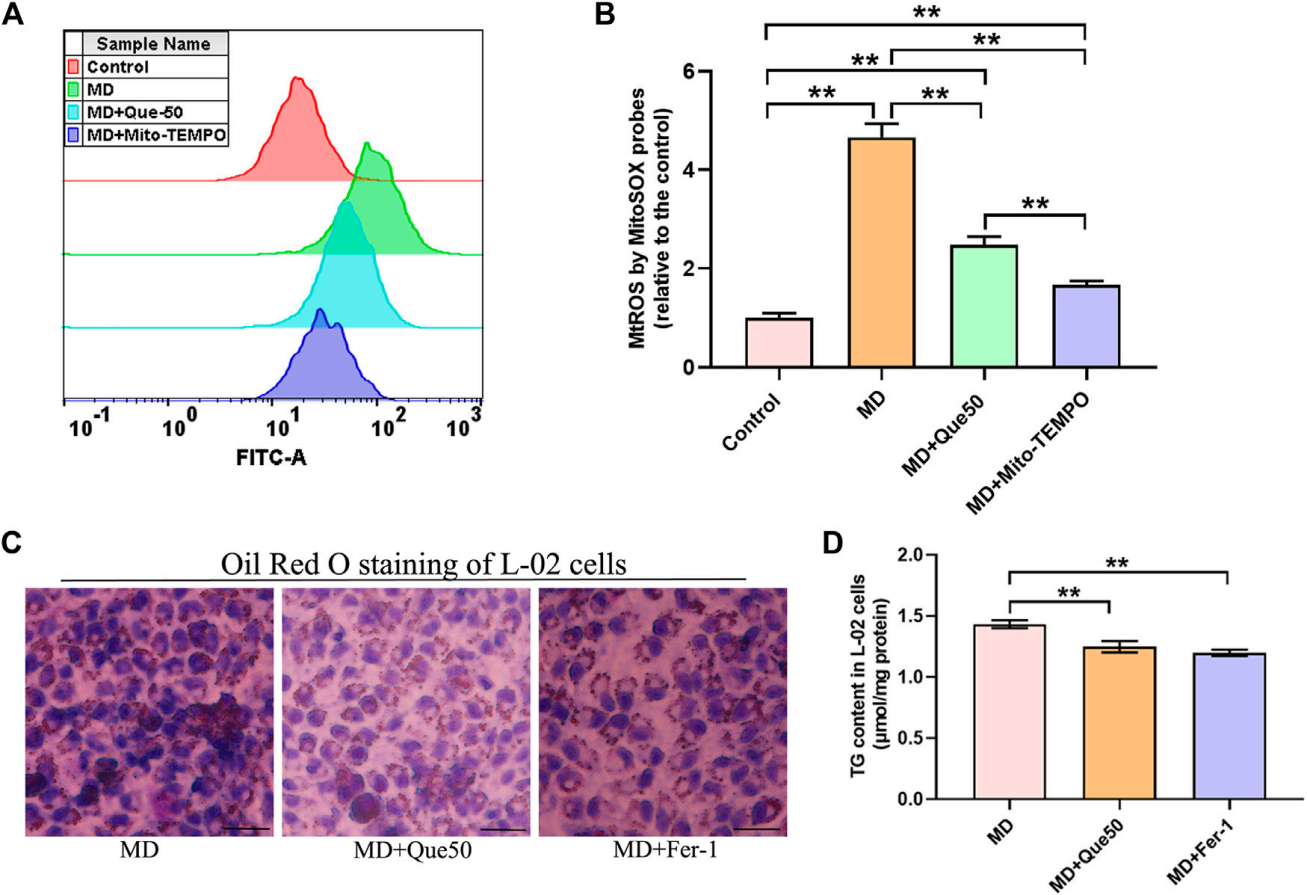
Que reduced MtROS and lipid deposition in steatotic L-02 cells (*n* = 3). **(A)** Representative fluorescence intensity images of MtROS by MitoSOX probes obtained by flow cytometry. **(B)** Flow cytometric analysis of fluorescence intensity. **(C)** Oil Red O staining of L-02 cells (scale bar = 50 μm). **(D)** TG content in L-02 cells. The data are expressed as the mean ± SD from three independent experiments. **, *p* < 0.01 and *, *p* < 0.05.

To explore the link between MtROS and ferroptosis in steatotic hepatocytes, we pretreated steatotic L-02 cells with Mito-TEMPO (the MtROS scavenger). Compared with the control group, the level of MtROS was significantly increased in the MD group (Figures 6A,B). As presented in Figures 6A,B, Que and Mito-TEMPO had similar effect on the reducing of MtROS in steatotic hepatocytes. To further explore the role of ferroptosis in lipid droplet accumulation and lipid peroxidation in steatotic hepatocytes, the ferroptosis specific inhibitor Fer-1 was used. As shown in Figures 6C,D, the lipid droplet accumulation and the TG content in steatotic L-02 cells was reduced by Que treatment and Fer-1. We next compared the effects of Que with Mito-TEMPO and Fer-1 on lipid peroxidation and ferroptosis in steatotic hepatocytes. We found that Que exerted similar effects to these of Mito-TEMPO and Fer-1 in reducing the level of lipid peroxidation and inhibiting ferroptosis in steatotic hepatocytes (Supplementary Figures S1, S2). Together, these data indicated that ferroptosis was involved in lipid accumulation and cellular ferroptosis in steatotic L-02 cells was mediated by MtROS, which was inhibited by Que treatment.

## Discussion

The present study demonstrated that long-term HFD consumption increased the lipid accumulation and lipid peroxidation, and triggered ferroptotic cell death, ultimately leading to hepatic injury. Mechanistically, we revealed that HFD-induced ferroptotic cell death depended on MtROS. Moreover, Que improved HFD-induced hepatic lipotoxicityby inhibiting MtROS-mediated ferroptosis.

Feeding animals a HFD usually causes obesity and metabolic disorders such as NAFLD. Many studies have indicated that Que, which is a kind of flavonoid, has a beneficial function in NAFLD rodent models (Surapaneni and Jainu, 2014; Kim et al., 2015; Porras et al., 2017; Porras et al., 2019) and in NAFLD patients (Pasdar et al., 2020), but the underlying mechanisms are still unclear. In this study, we observed that HFD consumption increased body weight gain and liver weight and caused lipid metabolic disorder, liver injury, and lipid accumulation. In line with these previous studies, our present data suggested that Que showed beneficial effects on HFD-induced NAFLD, such as altering the levels of serum lipids, liver injury, lipid accumulation and hepatic steatosis. Ferroptosis, a newly identified type of “programmed necrosis” characterized by lipid peroxidation in an iron-dependent manner (Dixon et al., 2012; Chen et al., 2020), has been confirmed to be involved in the pathological processes of various diseases (Fang et al., 2019; Martin-Sanchez et al., 2020; Mahoney-Sanchez et al., 2021). Recently, ferroptosis was proven to affect the progression of NASH by modulating lipid peroxidation in a methionine/choline-deficient diet-induced NASH mouse model (Ning et al., 2020). Previous studies have reported that Que has the ability to alleviate pancreatic β cell injury in type 2 diabetes and acute kidney injury by inhibiting ferroptosis (Li D. et al., 2020; Wang et al., 2021), but whether Que can inhibit ferroptosis to improve NAFLD has not been studied. In the present study, we found that HFD caused lipid peroxidation, ferroptosis and lipid accumulation in the liver, and Que treatment obviously ameliorated these changes in HFD-induced NAFLD model mice. Collectively, Que supplementation against NAFLD may target lipid peroxidation and lipid accumulation by inhibiting hepatic ferroptosis.

To further clarify the mechanism by which ferroptosis is involved in NAFLD, a steatotic hepatocyte model was established with L-02 cells, as evidenced by aberrant lipid accumulation changes. To further verify the effects of Que on ferroptosis and lipid peroxidation in steatotic L-02 cells, we quantified the TG content, the iron content, the expression of ferroptosis-related proteins and lipid peroxidation markers (4-HNE and GSH/GSSG). Our data first revealed that Que treatment inhibited hepatic ferroptosis and reduced lipid peroxidation and lipid accumulation *in vitro*.

Mitochondria are cytosolic organelles that regulate ATP production to provide energy for cellular metabolic homeostasis (Picard et al., 2016). Hepatic mitochondria in NAFLD are altered structurally and molecularly (Einer et al., 2018). Mitochondrial oxidative function in hepatocytes reportedly plays a vital role in the development of NAFLD (Shum et al., 2020). In addition, many studies in animal model cell culture and human patients have reported MtROS production and its causal role in NAFLD (Simoes et al., 2018). However, the signaling pathways that link mitochondrial dysfunction to the progression stages of NAFLD remain unclear. Under the condition of hepatic lipid accumulation, the liver attempts to recover from fat metabolic disorder, and mitochondrial fatty acid oxidation and the introduction of the tricarboxylic acid cycle are enhanced (Sunny et al., 2011). Mitochondria play an important role in ferroptosis (Jelinek et al., 2018), and several important metabolic processes in mitochondria (such as mitochondrial fatty acid oxidation, tricarboxylic acid cycle, and glutaminolysis) are involved in ferroptosis (Gao et al., 2019). Moreover, increased mitochondrial fatty acid oxidation and tricarboxylic acid cycle stimulation in NAFLD results in overeduction of the respiratory complexes, which promotes superoxide production (Aharoni-Simon et al., 2011; Simoes et al., 2018). In this study, we observed that MtROS was increased in steatotic hepatocytes, which is in accordance with a previous study (Kim et al., 2020). In an *in vitro* study, we found that mito-TEMPO and Que significantly reduced the production of MtROS in FFA mixture-treated steatotic hepatocytes, suggesting that Que has the ability to reduce hepatic MtROS. A previous study confirmed that excessive MtROS can trigger ferroptosis (Wei et al., 2020). *In vitro*, triggered hepatic ferroptosis and increased lipid peroxidation were observed in steatotic hepatocytes. Moreover, mito-TEMPO and Que apparently reduced hepatic ferroptosis and lipid peroxidation in steatotic hepatocytes. These results indicated that Que inhibits ferroptosis and lipid peroxidation by reducing hepatic MtROS. Importantly, lipid ROS promote NASH by boosting lipid droplet formation (Li X. et al., 2020). To date, the effect of hepatic ferroptosis on lipid deposition has not been clarified. Ferroptosis is associated with the processes of lipid synthesis, storage and degradation (Li and Li, 2020). Moreover, a previous study confirmed that GPX4 (a key regulator of ferroptosis) promotes lipid deposition in L-02 cells (Lu et al., 2021). In this study, our data indicate that inhibition of ferroptosis by Fer-1 (an inhibitor of ferroptosis) and Que decreased both lipid peroxidation and lipid droplet accumulation. Thus, increased ferroptosis is involved in hepatic lipid droplet accumulation. Taken together, our findings prove that HFD treatment increases hepatic ferroptosis, lipid peroxidation and lipid droplet accumulation and further demonstrate that MtROS are one of the first hits that cause NAFLD progression, which is diminished by Que treatment.

In conclusion, we first demonstrated that HFD consumption caused hepatic lipid peroxidationand lipid droplet accumulation by triggering ferroptosis in the liver. We also indicated that inhibiting MtROS-mediated ferroptosis by Que improved HFD-induced hepatic lipotoxicity and lipid accumulation. Hence, hepatic ferroptosis may be a new therapeutic target for HFD-induced metabolic liver disease.

## Data Availability

The datasets presented in this study can be found in online repositories. The names of the repository/repositories and accession number(s) can be found in the article/Supplementary Material.
