# Supplementary material for: Targeting Mitochondrial ROS-Mediated Ferroptosis by Quercetin Alleviates High-Fat Diet-Induced Hepatic Lipotoxicity
Source: Front Pharmacol. 2022 Apr 12;13:876550. doi: 10.3389/fphar.2022.876550 (PMC9039018; doi:10.3389/fphar.2022.876550)
Supplement: Supplementary file 1 [file DataSheet1.docx]

Supplementary Material

# Supplementary Figures

## Supplementary Figure 1


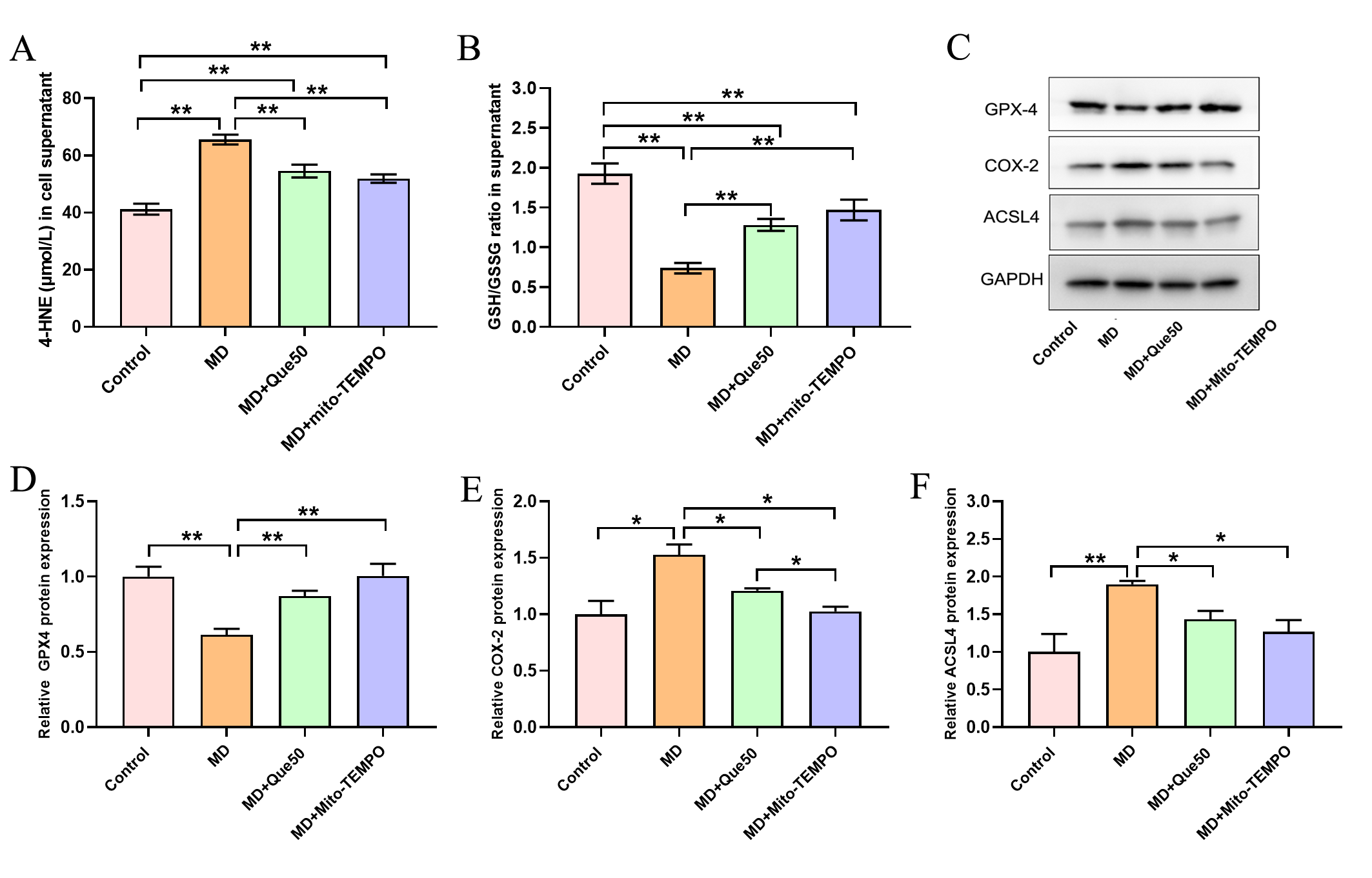


**Figure S1.** Effects of Que and Mito-TEMPO on lipid peroxidation and ferroptosis in steatotic L-02 cells. (A-B) The relative contents of 4-HNE and GSH/GSSG in L-02 cells. (C) Representative Western blot images. (D-F) Quantitative analysis of ferroptosis-related proteins (GPX4, COX-2 and ACSL4). The data are expressed as the mean ± SD from three independent experiments. **, *P*<0.01 and *, *P*<0.05.

## Supplementary Figure 2


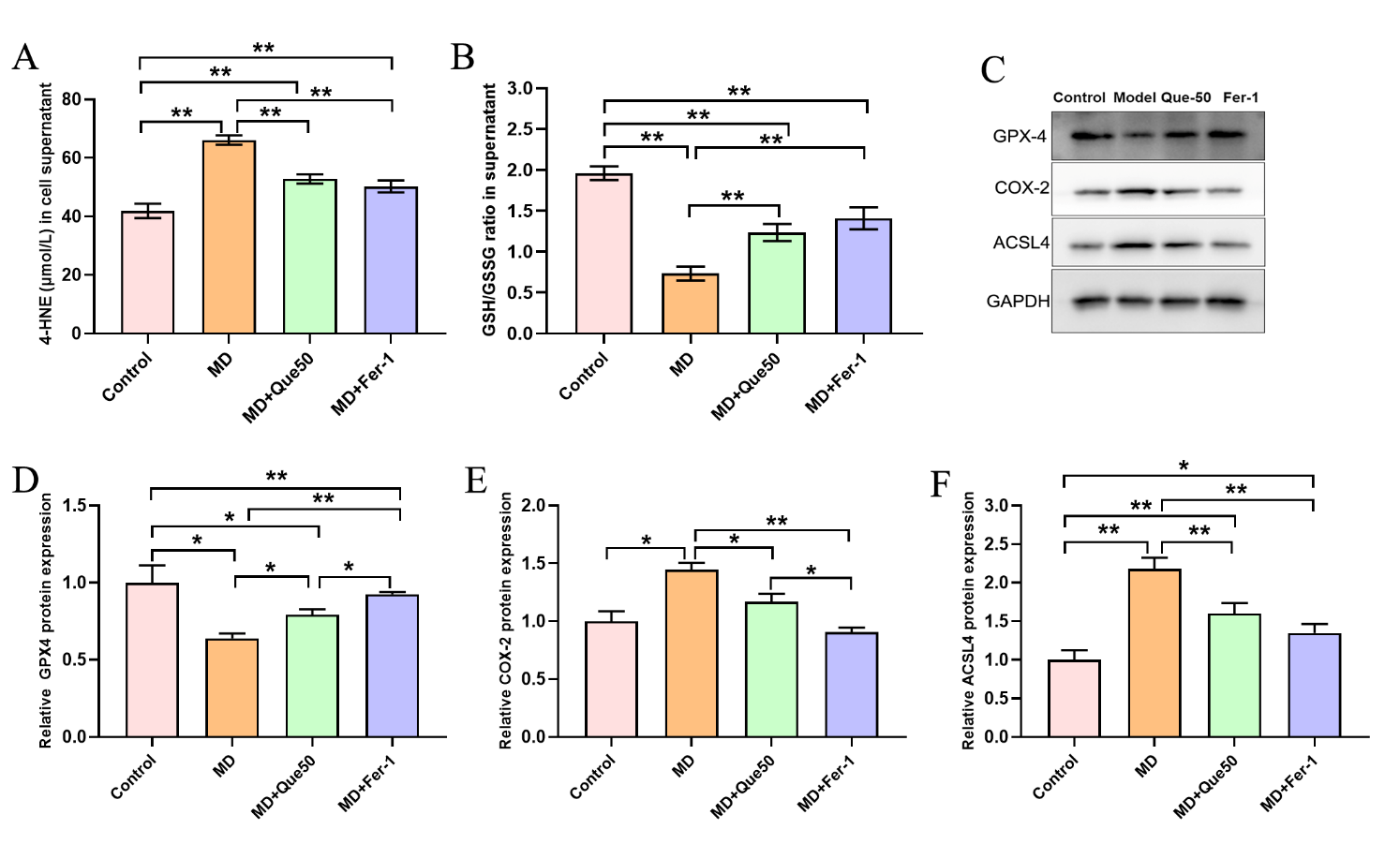


**Figure S2.** Effects of Que and Fer-1 on lipid peroxidation and ferroptosis in steatotic L-02 cells. (A-B) The relative contents of 4-HNE and GSH/GSSG in L-02 cells (n=3). (C) Representative Western blot images. (D-F) The expression of ferroptosis-related proteins (GPX4, COX-2 and ACSL4) in L-02 cells (n=3). The data are expressed as the mean ± SD from three independent experiments. **, *P*<0.01 and *, *P*<0.05.
